# Incidence rate, clinical profile, and outcomes of COVID-19 in adults with non-cystic fibrosis bronchiectasis

**DOI:** 10.36416/1806-3756/e20240258

**Published:** 2025-01-10

**Authors:** Cristiane Christ Camargo, Fernanda de Miranda Schmitz, Letícia Bauer Jacobsen, Bruna Ziegler, Paulo de Tarso Roth Dalcin

**Affiliations:** 1. Programa de Pós-Graduação em Ciências Pneumológicas, Universidade Federal do Rio Grande do Sul - UFRGS - Porto Alegre (RS) Brasil.; 2. Faculdade de Medicina, Universidade Federal do Rio Grande do Sul - UFRGS - Porto Alegre (RS) Brasil.; 3. Serviço de Fisioterapia, Hospital de Clínicas de Porto Alegre, Universidade Federal do Rio Grande do Sul - UFRGS - Porto Alegre (RS) Brasil.

## TO THE EDITOR:

The clinical outcomes of SARS-CoV-2 infection (COVID-19) range from asymptomatic cases to severe illness and death.^1^ Advanced age and comorbidities such as obesity, cardiovascular disease, pulmonary disease, certain types of cancer, and diabetes^2^ ^,^ ^3^ are risk factors for severe disease. Bronchiectasis, defined as an abnormal and irreversible dilatation of the bronchi, results in chronic inflammation of the lower airways and deterioration of lung function.^4^ Consequently, patients with non-cystic fibrosis (CF) bronchiectasis and SARS-CoV-2 infection are currently considered to be at an increased risk of developing severe manifestations of COVID-19. However, there are currently limited data on the profile of patients with non-CF bronchiectasis diagnosed with COVID-19 in Brazil, as well as on the incidence of COVID-19 in such patients. The objective of the present study was to describe the cumulative incidence of SARS-CoV-2 infection in patients with non-CF bronchiectasis monitored at the *Hospital de Clínicas de Porto Alegre* (HCPA), in the city of Porto Alegre, Brazil, during the two years of COVID-19 pandemic, as well as the clinical characteristics and outcomes of these patients. 

This was a retrospective study analyzing the incidence rate, clinical course, and outcomes of confirmed cases of COVID-19 in a cohort of adults with non-CF bronchiectasis. The study was approved by the Research Ethics Committee of the HCPA via *Plataforma Brasil* (Brazilian National Research Ethics Committee Database; Protocol no. 4.125.633). Written informed consent was obtained at recruitment. The study complied with the Declaration of Helsinki and the Brazilian government regulations. 

We enrolled 31 patients between April 30, 2020 and April 29, 2022. Inclusion criteria were adults with a diagnosis of non-CF bronchiectasis monitored at the HCPA during the COVID-19 pandemic. The diagnosis of non-CF bronchiectasis was based on CT imaging criteria.^5^

Clinical and demographic data were collected by reviewing the electronic medical records of the patients. The primary outcome of the study was the cumulative incidence of COVID-19 in the first and second years of study. The cases of COVID-19 were identified through telephone interviews and medical record review. Diagnostic criteria for COVID-19 were a positive real-time RT-PCR result from a nasopharyngeal swab, CT findings consistent with COVID-19, a clinical diagnosis of COVID-19 in a hospital setting, or any combination of the three. The clinical course of COVID-19 was rated on the WHO Ordinal Scale for Clinical Improvement.^6^

Data analysis was performed with the IBM SPSS Statistics software package, version 22.0 (IBM Corporation, Armonk, NY, USA). The sample size equaled the number of incident cases of COVID-19 during the study period. Data normality was examined with quantile-quantile plots and the Shapiro-Wilk test. Qualitative data were expressed as number of cases and proportion, and quantitative data were expressed as mean ± standard deviation or median and interquartile range. Categorical comparisons were performed with the chi-square test with Yates’ correction (when appropriate) or Fisher’s exact test. Continuous variables were compared by means of a t-test or the Wilcoxon-Mann-Whitney test. Cumulative incidence was calculated as the number of new cases of COVID-19 divided by the total number of individuals at risk for the study period (two years). The annual cumulative incidence of COVID-19 in the state of Rio Grande do Sul, Brazil, was also calculated, being adjusted for age.^7^ ^-^ ^9^ The chi-square test of independence was used in order to compare the annual cumulative incidence of COVID-19 between the study population and the general population. 

Of the 31 patients enrolled in the study, 5 were diagnosed with COVID-19: 2 in the first year of study and 3 in the second. The mean age of the patients was 39.4 years, 71% were female, and 93.5% were White. Most of the patients had bronchiectasis of uncertain etiology (48.4%), and 38.7% had a probable diagnosis of ciliary dyskinesia. Chronic infection with *Pseudomonas aeruginosa* was identified in 82.8% of the patients. The mean percent predicted FEV_1_ was 50.1 ± 24.1%, and the mean six-minute walk distance was 444.18 ± 81.4 m. Vaccination against COVID-19 began in May of 2021. Approximately 68% of the patients received three doses of COVID-19 vaccine, and 29% received two (Table 1). 

**Table 1:** Baseline patient data and comparison between patients with and without COVID-19.^a^

	Total N = 31	With COVID-19 n = 5	Without COVID-19 n = 26	p
Age, years	39.4 ± 14.1	31.6 ± 8.6	40.9 ± 14.6	0.182
Sex				0.613
Female	22 (71)	3 (13.6)	19 (86.4)	
Male	9 (29)	2 (22.2)	7 (77.8)	
Ethnicity				1.000
White	29 (93.5)	5 (17.2)	24 (82.8)	
Non-White	2 (6.5)	0	2 (100)	
Diagnosis				0.816
Ciliary dyskinesia	12 (38.7)	2 (16.7)	10 (83.3)	
Kartagener syndrome	3 (9.7)	0	3 (100)	
Obliterative bronchiolitis	1 (3.2)	0	1 (100)	
Uncertain	15 (48.4)	3 (20)	12 (80)	
Age at diagnosis	27 (8-38)	19 (11.5-35.5)	27 (8-40)	0.957
BMI, kg/m^2^	21.9 ± 4.1	22.0 ± 2.9	21.9 ± 4.3	0.874
History of pneumothorax	1 (3.2)	0	1 (100)	1.000
History of massive hemoptysis (> 100 mL)	2 (6.5)	0	2 (100)	1.000
History of bronchial artery embolization	0	0	0	
History of ABPA	1 (3.2)	0	1 (100)	1.000
On the lung transplant list	2 (6.5)	0	2 (100)	1.000
Lung transplant recipient	0	0	0	
*Pseudomonas aeruginosa*	24 (82.8)	2 (8.3)	22 (91.7)	0.127
MSSA	6 (20.7)	1 (16.7)	5 (83.3)	1.000
MRSA	0	0	0	
NTM	0	0	0	
Use of inhaled colistimethate sodium	11 (35.5)	1 (9.1)	10 (90.9)	0.631
Inhaled aminoglycoside therapy	4 (12.9)	0	4 (100)	1.000
Use of azithromycin	23 (74.2)	3 (13)	20 (87)	0.583
FVC, % predicted	61.7 ± 21.2	77.7 ± 21.6	56.3 ± 18.9	0.019
FEV_1_, % predicted	50.1 ± 24.1	60.0 ± 24.9	44.1 ± 19.1	0.097
FEV_1_/FVC, %	78.2 ± 14.9	75.2 ± 12.8	77.5 ± 15.2	0.843
6MWD, m	444.2 ± 81.4	503.5 ± 39.2	434.3 ± 82.8	0.117
SpO_2_, %	94.0 ± 2.5	93.2 ± 2.4	94.0 ± 2.4	0.500
No. of COVID-19 vaccine doses				0.242
2	9 (29)	3 (33.3)	6 (66.7)	
3	21 (67.7)	2 (9.5)	19 (90.5)	
None	1 (3.2)	0	1 (100)	
Deaths	3 (9.7)	0	3 (100)	1.000

ABPA: allergic bronchopulmonary aspergillosis; MSSA: methicillin-susceptible *Staphylococcus aureus*; MRSA: methicillin-resistant *Staphylococcus aureus*; NTM: nontuberculous mycobacteria; and 6MWD: six-minute walk distance. ^a^Data presented as n (%), mean ± SD, or median (IQR). *Chi-square test for categorical variables. ^†^Student’s t-test or Mann-Whitney U test for continuous variables.

The annual cumulative incidence of COVID-19 was 6.4% in the first year of study and 9.6% in the second. In the state of Rio Grande do Sul, there were 2.384.504 confirmed cases of SARS-CoV-2 infection on April 29, 2022, with an age-adjusted annual cumulative incidence of approximately 13% in the first year of study and 16% in the second.^7^ ^-^ ^9^ We found that the annual cumulative incidence of COVID-19 was not significantly different between the patients with non-CF bronchiectasis and the general population in the first and second years of study (p = 0.091 and p = 0.238, respectively). The fact that the cumulative incidence of COVID-19 was low in our cohort may be due to underreporting of COVID-19 cases in the first year of study, given that diagnostic tests were restricted to symptomatic cases with more severe respiratory symptoms. Moreover, people with chronic pulmonary diseases promptly adhered to respiratory protection measures, social distancing, and mask use. 

**Figure 1:**
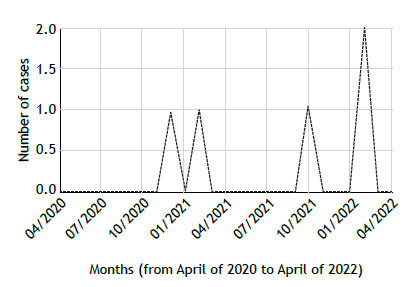
Distribution of COVID-19 cases in the study period.

The risk of SARS-CoV-2 infection did not differ between the study population and the general population in the first year of study (OR = 0.61; 95% CI, 0.31-1.20 vs. OR = 1.42; 95% CI, 1.01-2.00) or in the second (OR = 0.76; 95% CI, 0.45-1.26 vs. OR = 1.25; 95% CI, 0.89-1.76). The distribution of COVID-19 cases in the study period is presented in Figure 1. With regard to the clinical characteristics of patients, those with COVID-19 had higher percent predicted FVC than did those without COVID-19 (77.7 ± 21.6% vs. 56.3 ± 18.9%; p = 0.019), suggesting mild lung function impairment before SARS-CoV-2 infection. There were no differences between the two groups for the other variables. The most common symptoms at diagnosis of COVID-19 were myalgia, arthralgia, or both (in 80.0%); fever (in 80.0%); fatigue (in 60%); and cough (in 60%). All of the patients with SARS-CoV-2 infection had mild COVID-19 (a score of 1 or 2 on the WHO Ordinal Scale for Clinical Improvement) and required no hospitalization or ventilatory support, recovering completely from the infection. 

This study has potential limitations. It was conducted in a single medical center and included a relatively small sample, thus limiting its statistical power. The study had a retrospective design and used electronic medical record data, which are not likely to be as complete and accurate as prospective study data. Additionally, during the first phase of the COVID-19 pandemic, diagnostic tests were restricted to symptomatic cases with more severe respiratory symptoms, with the actual infection rate possibly being underestimated. 
